# Co-administration of Shexiang Baoxin Pill and Chemotherapy Drugs Potentiated Cancer Therapy by Vascular-Promoting Strategy

**DOI:** 10.3389/fphar.2019.00565

**Published:** 2019-05-24

**Authors:** Liu-qing Yang, Ru-yi Li, Xi-yan Yang, Qian-fei Cui, Fei-yun Wang, Guo-qiang Lin, Jian-ge Zhang

**Affiliations:** ^1^Innovation Research Institute of Traditional Chinese Medicine, Shanghai University of Traditional Chinese Medicine, Shanghai, China; ^2^School of Pharmaceutical Sciences, Zhengzhou University, Zhengzhou, China; ^3^Department of Respiratory and Critical Care Medicine, National Key Clinical Specialty, Xiangya Hospital, Central South University, Changsha, China

**Keywords:** cancer treatment, Shexiang Baoxin Pill, combination therapy, vascular promotion, drug delivery

## Abstract

Effective delivery of chemotherapeutic agents to tumors is a critical objective of improved cancer therapy. Traditional antiangiogenic therapy aims at eradicating tumor blood vessels, but the subsequently reduced blood perfusion may limit the drug amount delivered into the tumor and potentially lead to tumor hypoxia, which has been proved to be unable to meet the therapeutic expectations. “Shexiang Baoxin Pill” (SBP) is a well-known traditional Chinese medicine (TCM) used in clinical treatment of cardiovascular diseases, which has the pharmacological effect of pro-angiogenesis demonstrated recently. In this study, we disclosed our finding that SBP could enhance the effective treatment performance of gemcitabine (GEM) while minimizing the toxic side effects caused by GEM. Mechanistically, SBP increased tumor angiogenesis, blood perfusion, vascular permeability, and vessel dilation, which subsequently favored the delivery of GEM to the tumor lesion. Moreover, combined treatment with SBP and GEM could modify tumor microenvironment and consequently overcome multidrug resistance, and this combination therapy is also suitable for combination of SBP with some other chemotherapeutic drugs as well. These results suggest that combining SBP with chemotherapeutic agents achieves better treatment efficiency, which can open an avenue for expanding the combined treatment of anti-cancer chemotherapeutic drugs with TCM.

## Introduction

Anti-angiogenesis is a targeted therapeutic strategy and was once considered to be an effective treatment for cancers (Wu et al., 2018). In the past decades, several drugs have been developed on promoting vascular regression and tumor starvation to inhibit tumor growth such as angiogenesis inhibitors, vascular disrupting agents and so on (Kong et al., 2017). Nonetheless, the approach of eradicating tumor vasculature is not as effective as expected in that it may lead to odds of tumor hypoxia and drug resistance (Ebos et al., 2009; El-Kenawi and El-Remessy, 2013; Jain, 2014). Interestingly, antiangiogenic treatment can reduce blood vessel density to some extent, leading to vascular temporary normalization and subsequently improving blood perfusion and releasing of chemotherapy drugs to tumor tissue as well as enhancing the efficacy of chemotherapeutics (Maes et al., 2014). However, to reach this effectiveness, it requires the choice of optimal doses and time to use, otherwise, the tumor vasculature may close the therapeutic window due to its excessive suppression (Rolny et al., 2011; Van der Veldt et al., 2012). Thus, anti-angiogenic drugs are difficult to apply in clinical practice whether or not they are used alone or in combination with chemotherapeutic agents. Vascular disrupting agents aim at inducing tumor blood flow shutdown rapidly and selectively, resulting in massive necrosis (Siemann, 2011). Nevertheless, they may potentially enhance tumor hypoxia and elevated tumor metastasis, thus failing to meet the therapeutic expectations (Palumbo et al., 2011). Recent research has reported that promoting tumor angiogenesis and increasing blood flow would potentially improve the effectiveness of anti-tumor therapy (Bridges and Harris, 2015; Villanueva, 2015; Wong et al., 2015, 2016; Yin et al., 2017). This strategy for treating tumors is radically different from the previous methods and is called “vascular promotion therapy” which at the best of our knowledge has been rarely studied so far.

Shexiang Baoxin Pill (SBP) is a common drug for chest pain or discomfort caused by coronary heart disease (CHD) in China, which is derived from the prescription of the ancient Suhexiang Pill recorded in Prescriptions of the Bureau of Taiping People's Welfare Pharmacy (Cen et al., 2017; Fang et al., 2017; Wei et al., 2018). SBP is a traditional Chinese medicine preparation comprised of seven substances common components of Chinese medicine, including Moschus, Calculus Bovis, Radix Ginseng, Styrax, Cortex Cinnamomi, Venenum Bufonis, and Borneolum Syntheticum, the representative compounds and structures of each medicinal material are shown in Table 1. With the deepening of research, chemical fingerprinting and quantitative analysis of non-volatile and volatile compounds of SBP have been reported(Yan et al., 2006). More than 70 non-volatile and 40 volatile compounds have been identified in SBP by using chromatography and mass spectrometry techniques (Liu et al., 2015; Lv et al., 2017). The potential active ingredients in Shexiang Baoxin Pills were analyzed by GC/MS and UPC2-MS/MS methods, the average content of which was: Muscone (65.54 μg/ml, 0.09%); Ginsenoside Re (41.22 μg/ml, 0.05%); Ginsenoside Rb1(151.94 μg/ml, 0.20%); Cinnamaldehyde (17.69 μg/ml, 0.02%); Cholic acid (75.08 μg/ml, 0.10%); Bufalin (31.07 μg/ml, 0.04%); Benzyl benzoate (278.46 μg/ml, 0.36%); Borneol (807.15 μg/ml, 2.0%) (Yan et al., 2006, 2009; Zhang et al., 2018). Moreover, researchers used chromatographic-tandem mass spectrometry and plasma-based chemistry to screen potential bioactive components after oral SBP (Jiang et al., 2009; Lu et al., 2018). The relevant results indicate that there are four volatile components in the plasma after oral administration of SBP in rats, including isobornyls, borneol, musk ketone, and cinnamaldehyde (Chang et al., 2014). Cinnamon aldehyde, a small molecule enriched in cinnamon, has been shown to be a potent vasodilator that activates transient receptor potential channels (TRPV1 and TRPA1) involved in nociception (Yanaga et al., 2006; Szallasi et al., 2007; Sui et al., 2010). It induces angiogenic activity and accelerates wound healing (Yuan et al., 2018). SBP can alleviate arterial obstructive disease by promoting angiogenic activity (Zhang et al., 2017; Guo et al., 2018). SBP confers enhanced angiogenic capacity to EPCs and is thought to be an agonist of angiogenesis by inducing expression of endothelial nitric oxide synthase (eNOS) and subsequent production of nitric oxide (NO) (Li et al., 2015).

**Table 1:** Composition of Shexiang Baoxin Pill.

**Medicinal materials**	**Representative compounds**	**Representative compounds 2D structure**	**Therapeutic effects**
Artificial moschus	**Muscone**	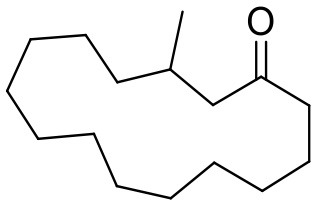	Promotes blood circulation and relieves blood stasis, alleviates pain, and subdues swelling.
Cortex cinnamomi	**Cinnamaldehyde**, Cinnamic acid	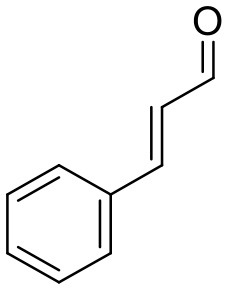	Dilates blood vessels, stimulates angiogenesis, promotes blood circulation, and wound healing. Displays anti-oxidative, anti-microbial, anti-cardiovascular disorders, and anti-diabetic effects.
Borneolum	**Borneol**, Isoborneol	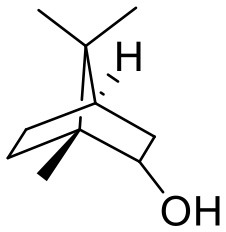	Dilates blood vessels and alleviates pain, anti-inflammatory.
Radix ginseng	**Ginsenoside Re**, Ginsenoside Rb1	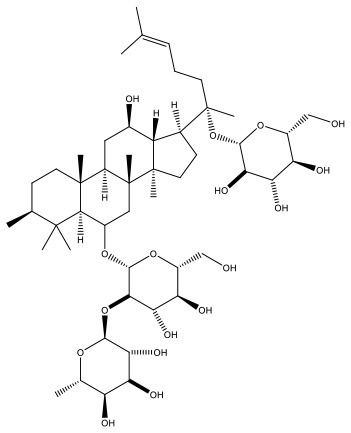	Disperses cold and alleviates pain, tranquilizes the mind and improves intelligence.
Calculus bovis	**Cholic acid**, Ursodeoxycholic acid, Chenodeoxycholic acid	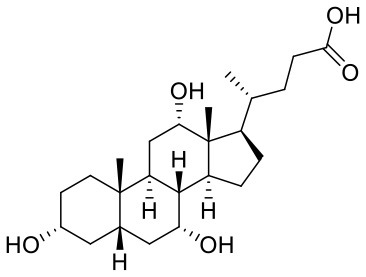	Clears away heat and toxins, resolves phlegm to clear orifice and induces resuscitation, decreases the production of cholesterol and reduces hypertriglyceridemia.
Styrax	**Benzyl benzoate**	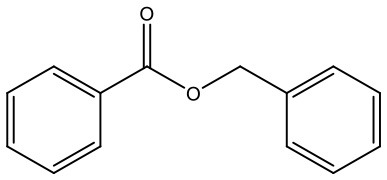	Disperses cold and alleviates pain, resolves phlegm. Displays anti-thrombotic and anti-inflammatory effects.
Venenum Bufonis	**Bufalin**, Cinobufagin Gamabufotalin	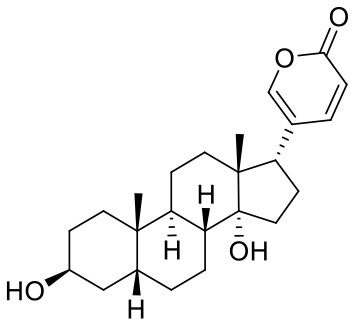	Clears away heat and toxins, alleviates pain and refreshes the mind. Displays anti-inflammatory and cardiotonic effects.

Gemcitabine (GEM) is one of the most commonly employed anti-cancer agents and is frequently administered against non-small cell lung cancer (NSCLC). However, with the increasing dosage of the drug, its inherent severe side effects and adverse reactions unavoidably appeared (Katircibasi and Eken, 2017), which limit the long-term clinical usage.

Therefore, we hypothesized that it could enhance the treatment performance of GEM in the presence of SBP, whilst minimizing the adverse effects. To validate this hypothesis, SBP combined GEM was tested in the treatment of Lewis lung carcinoma (LLC) tumor-bearing C57BL/6 mice in comparison with GEM alone to explore the anti-tumor effect *in vivo*. Following by that, the investigation of the effect of SBP on tumor vasculatures and tumor microenvironment was conducted, and changes of tumor angiogenesis, blood supply, vascular permeability, and tumor hypoxia degree were detected in mice treated with the combination of SBP and GEM afterwards. Next, the content of GEM in the tumor tissue was detected by High Performance Liquid Chromatography (HPLC) method. Moreover, the difference between combined therapy and individual medicine was performed for comparison.

## Materials and Methods

### Materials

SBP was purchased from Shanghai Hutchison Pharmaceuticals (Z31020068, Shanghai, China). Gemcitabine (GEM), cisplatin (DDP), and Cyclophosphamide (CTX) were purchased from Jiangsu Haosen Pharmaceutical Co., Ltd. (Dalian, China). Nedaplatin (NDP) were purchased from Qilu Pharmaceutical Co., Ltd. (Shandong, China).

### Cell Lines and Animals

Murine LLC cells (FuDan IBS Cell Center, FDCC, China) were maintained in RPMI-1640 supplemented with 10% fetal bovine serum (FBS). The cell lines were incubated in a humidified atmosphere containing 5% CO_2_ at 37°C.

C57BL/6 mice (male, 6–8 weeks, 20–22 g) were purchased from the Experimental Animal Center of Shanghai University of Traditional Chinese Medicine (Shanghai, China). All experimental protocols were approved by the Ethical Committee of Shanghai University of Traditional Chinese Medicine and the approval number is PZSHUTCM190404001. The care of all animals was maintained under standard housing conditions.

### Animal Model Establishment

To establish the subcutaneous tumor models, C57BL/6 mice were anesthetized by sodium pentobarbital (1%) and given a slowly subcutaneous injection of 4 × 10^6^ LLC cells into the right posterior limb. Tumor sizes were measured by caliper and calculated by using the formula V = [length × (width) ^2^]/2. When the tumors reached the size of 100 mm^3^, the tumor-bearing mice model was successfully constructed and the drug was given.

### *In vivo* Antitumor Efficacy Assay

The antitumor activity *in vivo* by co-administration of SBP with GEM was first evaluated in LLC tumor-bearing mice. The mice were randomly divided into seven groups and treated separately with saline, SBP (32 mg/kg), GEM (35 mg/kg), GEM (200 mg/kg), SBP (16 mg/kg) combined with GEM (35 mg/kg), SBP (32 mg/kg) combined with GEM (35 mg/kg) and SBP (64 mg/kg) combined with GEM (35 mg/kg), respectively. SBP was administrated by intragastric injection once a day, and GEM by intraperitoneal (i.p.) injection every 3 days. The weight of the mice and tumor volume was measured every other day. At the end of the experiment, tumor tissues were collected and then fixed in 4% paraformaldehyde solution.

The LLC tumor-bearing mice in each group were administrated separately with saline, SBP, NDP (6 mg/kg), NDP (12 mg/kg), SBP combined with NDP (6 mg/kg), DDP (4 mg/kg), DDP (6 mg/kg), SBP combined with DDP (4 mg/kg), CTX (15 mg/kg), CTX (30 mg/kg), and SBP combined with CTX (15 mg/kg). SBP was treated by intragastric injection once a day at a dose of 32 mg/kg, and NDP, DDP, CTX by i.p. injection every 3 days. The tumor volume was measured every other day. The rate of tumor inhibition in each treatment was subsequently calculated based on the tumor volume at the end of experiment.

### Immunohistochemistry and Immunofluorescence Analysis

On the ninth day after administration, the mice were sacrificed and tumor tissues of mice were harvested for hematoxylin and eosin (H&E) staining and terminal transferase dUTP nick-end labeling (TUNEL) assay kit staining to measure the induced apoptosis. The tissue sections were stained by using Glut1 (Bioss, Beijing, China) and CAIX(Bioss, Beijing, China) antibody to display the hypoxia area. For detection of tumor desmoplasia, the α-SMA antibody (Abcam, UK), and the Picro-Sirius Red Stain Kit (Solarbio, Beijing, China) were used. Besides, immunofluorescence sections of LLC tumor tissue were stained using CD31 (Abcam, UK) antibody to show the vascular density. Positive signals were captured using a fluorescence microscope equipped with a camera (Nikon DS-Qi1MC, Nikon Corporation).

### Blood Perfusion

The LLC tumor-bearing mice with the tumor volume of ~100 mm^3^ were i.p. pretreated with saline or SBP (32 mg/kg) once a day (*n* = 5 each group). Seven days later, each mouse was intravenously injected with 100 μL of 2,000-kDa-isothiocyanate-fluorescein-labeled dextran (Sigma-Aldrich, Saint Louis, USA) at 120 mg/kg. Then, 20 min after the tracer was injected, the mice were anesthetized and the images were recorded using the *in vivo* imaging system (IVIS) with a 465 nm excitation wavelength and a 520 nm filter. After that, animals were sacrificed and the tumor tissues were excised for further visualization by *in vivo* imaging system. Tumors were snap frozen, sectioned, stained with CD31 and examined by confocal microscopy.

### Vascular Permeability

Extravascular diffusion was tested by injecting mice via the tail vein with 100 μL 70-kDa-isothiocyanate-fluorescein-labeled dextran (Sigma-Aldrich, Saint Louis, USA) at 100 mg/kg. Animals were sacrificed 30 min after dextran injection, and tumor tissues were carefully dissected, stained with CD31 and examined by confocal microscopy.

### Detection of Nitric Oxide (NO)

NO release in tumor tissue was investigated in LLC tumor-bearing mice. They were administered separately with normal saline, SBP (32 mg/kg), GEM (35 mg/kg), and SBP (32 mg/kg)/GEM (35 mg/kg). On the ninth day after administration, the mice were sacrificed and tumors were removed and immediately frozen at −80°C. NO releasing was studied by Nitrate/Nitrite colorimetric assay kit (Beyotime, Shanghai, China).

### HPLC Measurements of GEM Concentrations in Tumor

Tumor-bearing mice with the tumor volume of ~100 mm^3^ were given a treatment with either GEM or the combination of SBP with GEM. The experiments were carried out by i.p. injection with GEM at a dose of 35 mg/kg every three days and intragastric injection with SBP at a dose of 32 mg/kg every day. On the seventh day after treatment, at the predetermined time of 0.5, 1, 2, 3, 4, and 5 h after injection, mice were sacrificed and the tissues were homogenized in saline with W/V = 1/4 (W: tumor weight, V: saline volume). After 135 μL tissue homogenate was placed into 2 mL centrifuge tube, 1.2 mL Methanol and acetonitrile (1:9, v/v) was added to the tubes and mixed by vortex. After centrifugation for 10 min at 12,000 rpm, the supernatant was taken out and dried under nitrogen. Then, the contents of GEM in different tissues were measured by using HPLC.

### *In vivo* Toxicity Analysis

Healthy C57BL/6 mice (6 weeks, male) were randomly divided into three groups. Mice were given separately saline, Gem (200 mg/kg) or the combination of SBP (32 mg/kg) with GEM (35 mg/kg). At the end of experiment, all the mice were sacrificed, and blood, heart, liver, spleen, lung, and kidney were removed. Blood was used to measure the value of alanine aminotransferase (ALT), aspartate aminotransferase (AST), total cholesterol (TC), triglyceride (TG), urea (UREA) and routine blood test. The major organs were washed and then fixed in 4% paraformaldehyde solution to prepare H&E staining.

### Statistical Analysis

The collected quantitative data were expressed as mean ± standard deviation (SD). Statistics significance was analyzed by the Student's test and one way analysis of variance (ANOVA) test. Statistical significance was inferred at a value of ^*^P < 0.05, ^**^P < 0.01, ^***^P < 0.001.

## Results

### Treatment With Combination SBP and GEM Inhibits Lung Cancer Growth While Increasing Tumor Angiogenesis

**Figure 1:**
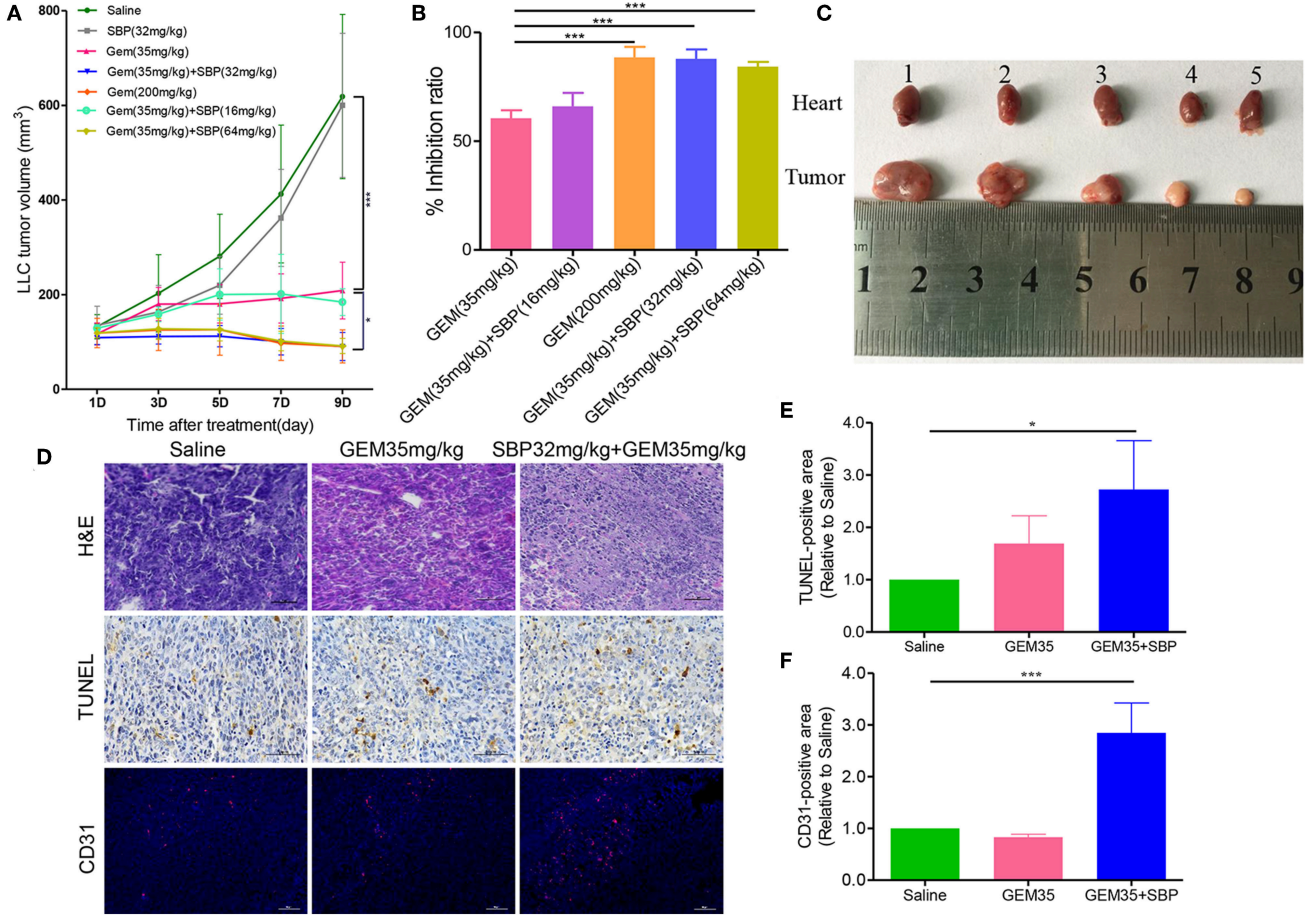
Effect of SBP combined with GEM on tumor growth and angiogenesis in Lewis lung carcinoma (LLC) tumor models. **(A)** Tumor volume curves of different treatment groups (*n* = 6–12 for each group). **(B)** Quantitative analysis of the tumor inhibition rate (*n* = 6–12 for each group). **(C)** The images of tumor tissues at treatment termination. (1) Saline; (2) SBP (32 mg/kg); (3) GEM (35 mg/kg); (4) GEM (200 mg/kg); (5) GEM (35 mg/kg)/SBP (32 mg/kg). **(D)** Representative images of H&E, TUNEL, and CD31-stained tumor sections. (Scale bar: 50 μm). **(E,F)** The quantitative analysis of TUNEL^+^ area and CD31^+^ area compared to the saline group. ^*^p < 0.05, and ^***^p < 0.001.

*In vivo* antitumor activity was evaluated in murine syngeneic LLC tumor-bearing mice. Seven groups of tumor growth curves are shown in Figure 1A. The results showed that, compared with the control group, all the treatments except the SBP (32 mg/kg) monotherapy exhibited anti-tumor activity to some degree. The anti-tumor treatment effect with Gem (35 mg/kg) was similar to the SBP (16 mg/kg)/GEM (35 mg/kg) group, with a weak effect in both groups. In contrast, the suppressed effects of SBP (32 mg/kg)/GEM (35 mg/kg) and SBP (64 mg/kg)/GEM (35 mg/kg) were of significance, comparable to those of the GEM (200 mg/kg) group. These data suggest that SBP can improve the anti-tumor efficacy of GEM even at a lower dose of 32 mg/kg. Similar findings were observed both in tumor inhibition rates and morphology (Figures 1B,C). There was 90.07% inhibition rate of tumor growth in the SBP (32 mg/kg)/GEM (35 mg/kg) treated group, which is markedly higher than the GEM (35 mg/kg) treated group (*P* < 0.001).

Next, cell apoptosis in xenograft was evaluated by H&E staining and TUNEL assay. The results of H&E staining showed characteristic apoptosis morphological changes, including condensed nuclei, cytoplasmic vacuolization and cell shrinkage in the tumor sections from treatment groups, especially in SBP(32 mg/kg)/GEM(35 mg/kg) treated group (Figure 1D). The TUNEL staining results showed that the recipe of SBP (32 mg/kg) combined with GEM (35 mg/kg) presented the best antitumor efficacy in LLC tumor model, as evidenced by the largest area of apoptosis (Figures 1D,E). In addition, analysis of lung tumor vasculature showed that treatment with SBP (32 mg/kg)/GEM (35 mg/kg) significantly promoted tumor blood vessel density (Figures 1D,F).

### SBP Enhances Blood Perfusion, Microvascular Permeability and Vessel Dilation

**Figure 2:**
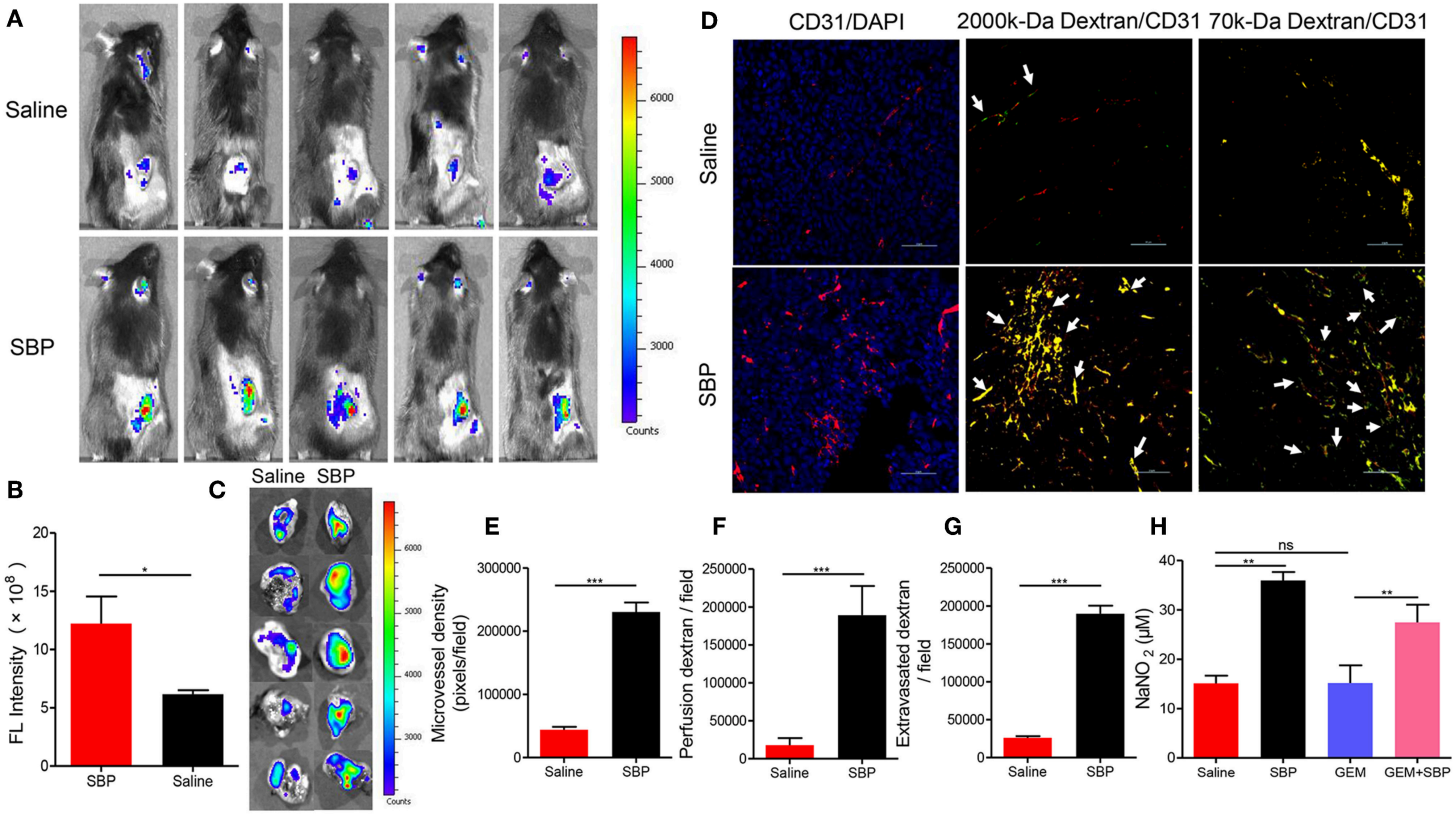
*In vivo* blood perfusion, microvascular permeability and vessel dilation. **(A)** *In vivo* fluorescence imaging at 20 min after 2,000-kDa-isothiocyanate-fluorescein-labeled dextran injection (*n* = 5 for each group). **(B)** The quantitative analysis of the fluorescence intensity in tumor tissues (*n* = 5 for each group). **(C)** *Ex vivo* fluorescence imaging at 20 min after 2,000-kDa-isothiocyanate-fluorescein-labeled dextran injection (*n* = 5 for each group). **(D)** Micrographs of CD31^+^ microvessels (red) in association with DAPI^+^ nuclei (blue in left panels), perfusion of 2,000-kDa dextran (green in middle panels), and leakiness of 70-kDa dextran (green in right panels). Arrows in middle panels indicate perfused tumor vessels. Arrowheads in right panels indicate leaked dextran signals. (Scale bar: 50 μm). **(E–G)** Quantification of CD31^+^ tumor vessels, perfusion of 2,000-kDa dextran, and extravasated 70-kDa dextran signals in LLC cancers (*n* = 5 for each group). **(H)** Calculation of NO expression levels in tumor tissues (*n* = 5 for each group). ^*^p < 0.05; ^**^p < 0.01; ^***^p < 0.001. ns, not significant.

In order to explore the possible mechanisms underlying the enhanced tumor inhibition afforded by SBP combined with GEM, the vascular function in these tumors was detected by *in vivo* fluorescence imaging and immunofluorescence. As displayed in Figures 2A,B, mice treated with SBP displayed a stronger fluorescent signal in the tumor area 20 min after injection with more than 2-fold (*P* < 0.05) signal strength compared with the saline group. Furthermore, the *ex vivo* images of tumors are shown in Figure 2C. Fluorescent signal of SBP treatment group was higher than the saline group. The frozen-section result revealed that micro-vasculatures in SBP-treated LLC tumor bearing mice showed distinctly increase of blood perfusion and angiogenesis (Figures 2D–F, *P* < 0.001 vs. saline group). Moreover, treatment of SBP markedly enhanced tumor vessels regarding leakage of 70-kDa dextran molecules (Figures 2D,G, *P* < 0.001 vs. saline group). These data clearly demonstrated that SBP treatment could effectively increase tumor blood perfusion and vascular permeability.

Then, the attention was turned to NO, an endothelium-derived relaxing factor, which plays a key role in vasodilatation of vasculature (Kim et al., 2017). It could be seen that the NO releasing from the mice treated with SBP was much higher than those incubated with saline (*p* < 0.001). Moreover, that from the mice treated with SBP (32 mg/kg)/GEM (35 mg/kg) was also significantly higher than those mice administrated with GEM (35 mg/kg) (Figure 2H, *p* < 0.01), indicating that SBP could stimulate the release of NO in tumors, thereby dilating blood vessels.

### SBP Increases Tumor Vascular Function and Intratumoral Drug Delivery

**Figure 3:**
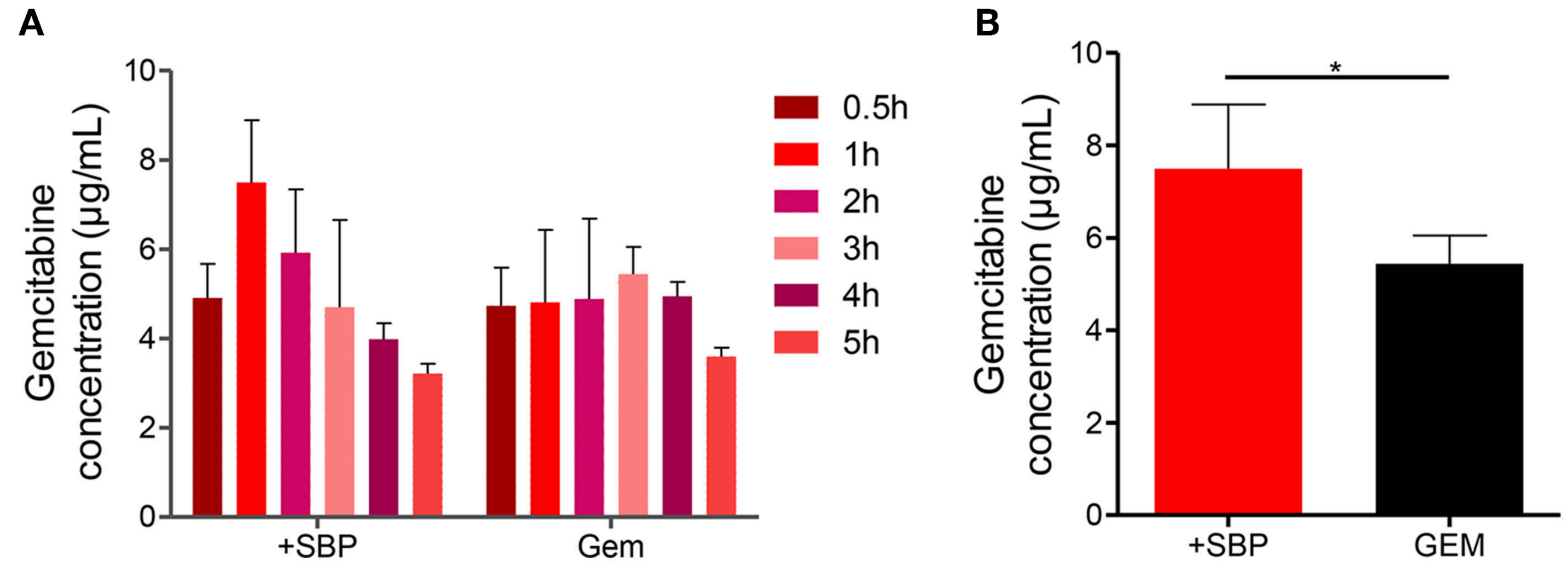
GEM accumulation in LLC tumor model after SBP treatment. **(A)** GEM content in tumor tissue for different time intervals and **(B)** GEM maximum content in tumor tissue (*n* = 3–5 for each group). ^*^p < 0.05.

Next, the attempt was given to investigate whether or not the increases in blood vessel density, permeability and perfusion together with vasodilation were sufficient to increase GEM delivery. HPLC analysis showed that the amount of intratumoral GEM, after intraperitoneal GEM injection, was increased significantly in mice treated with SBP (32 mg/kg)/GEM (35 mg/kg) in comparison with GEM (35 mg/kg) alone (Figures 3A,B, *P* < 0.01). These results illustrated that treatment with SBP at the dosage of 32mg/kg was capable of driving GEM delivery to tumors, which indeed strengthened the therapeutic efficacy of GEM. This might be an alternative regime to the conventional conflicts between reducing tumor vessel density and improving drug delivery.

### Tumor Microenvironment (TME) Modifications

**Figure 4:**
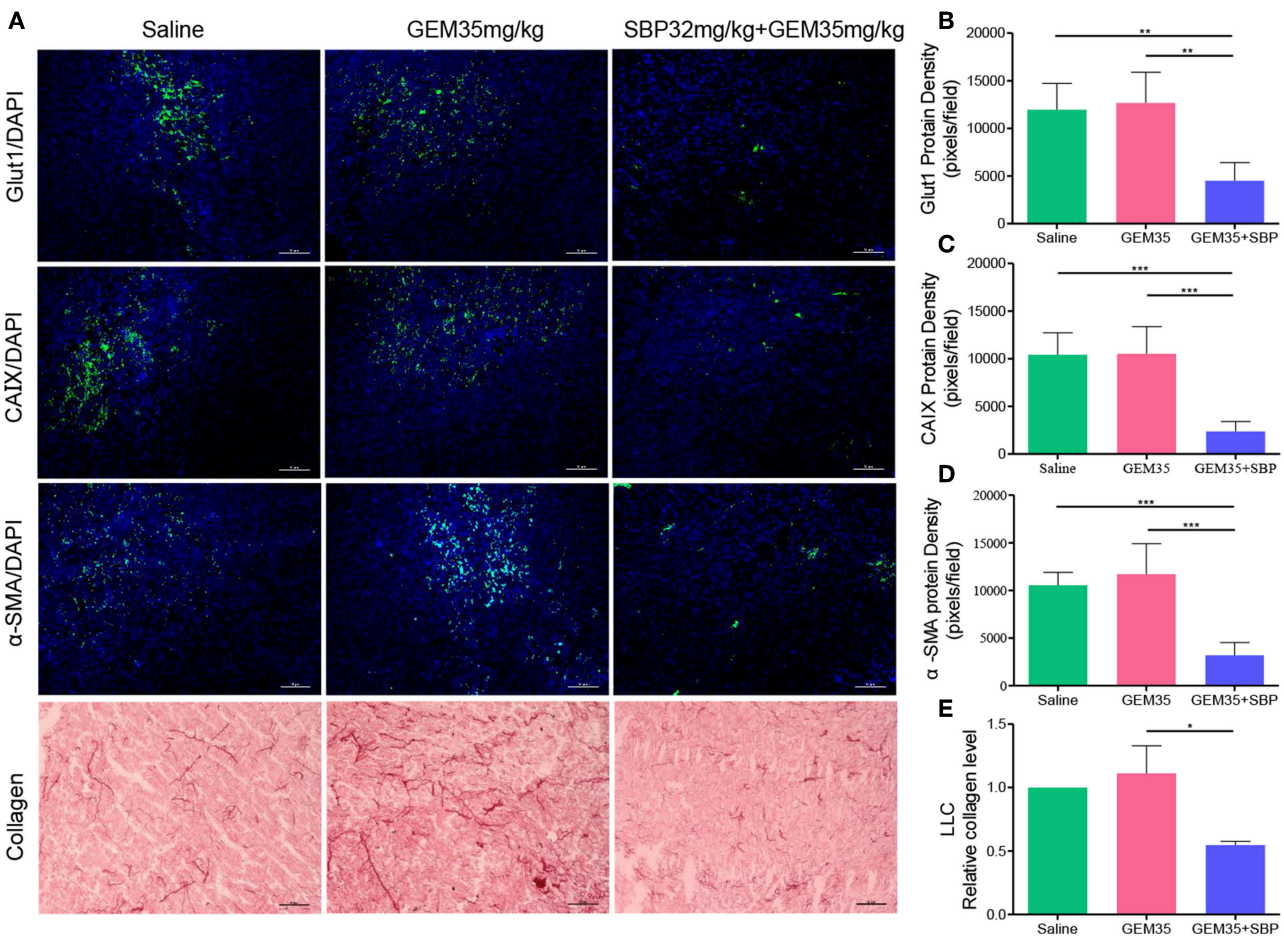
The effect of combination therapy with SBP and GEM on the tumor microenvironment in LLC tumor model. **(A)** Representative micrographs of Glut1^+^, CAIX^+^, α-SMA^+^, and collagen^+^ signals of Saline, GEM 35 mg/kg, and SBP 32 mg/kg/GEM 35 mg/kg treated mice. (Scale bar: 50 μm). **(B–E)** Quantification of Glut1^+^, CAIX^+^, α-SMA^+^, and cleaved collagen^+^ signals of Saline, GEM 35 mg/kg and SBP 32 mg/kg/GEM 35 mg/kg treated mice (*n* = 5 for each group). ^*^p < 0.05; ^**^p < 0.01; ^***^p < 0.001.

We next investigated the impact of SBP (32 mg/kg)/Gem (35 mg/kg) treatment on the TME. While hypoxic microenvironment is a typical characteristic of solid tumors, which is strongly correlated with cancer progression and chemoresistance (Martin et al., 2016; Gupta et al., 2017; Seleit, 2017). Angiogenesis has been reported to help in reducing tumor hypoxia (Wong et al., 2015). Thereby, tumor hypoxia area was evaluated through Glut1 and CAIX staining as shown in Figures 4A–C. Compared to saline and GEM (35 mg/kg), the hypoxia area was reduced more than one-third of that caused by treatment of SBP (32 mg/kg)/GEM (35 mg/kg) (*P* < 0.01), owing to the increased vessel dilation and density.

Tumor desmoplasia is characterized by increased collagen deposition and infiltration of α-SMA positive muscle fibroblasts (Huang et al., 2014). It is verified that tumor desmoplasia contributes to tumor progression and chemoresistance (Travis et al., 2011; Popper, 2016). High collagen and organized oriented deposition promote tumor cell migration (Huang et al., 2014). Immunofluorescence analysis of α-SMA showed that the staining of SBP (32 mg/kg)/GEM (35 mg/kg) group decreased by 69.82% compared with that of the saline group (Figures 4A,D, *P* < 0.001). In line with these observations, statistical analysis of collagen showed 54.73% reduction in staining in SBP (32 mg/kg)/GEM (35 mg/kg) treated tumors compared to the saline group (Figures 4A,E, *P* < 0.05). Analysis of these data indicated that treatment with SBP (32 mg/kg)/GEM (35 mg/kg) reduced tumor hypoxia and desmoplasia.

### Minimizing Adverse Side Effects

**Figure 5:**
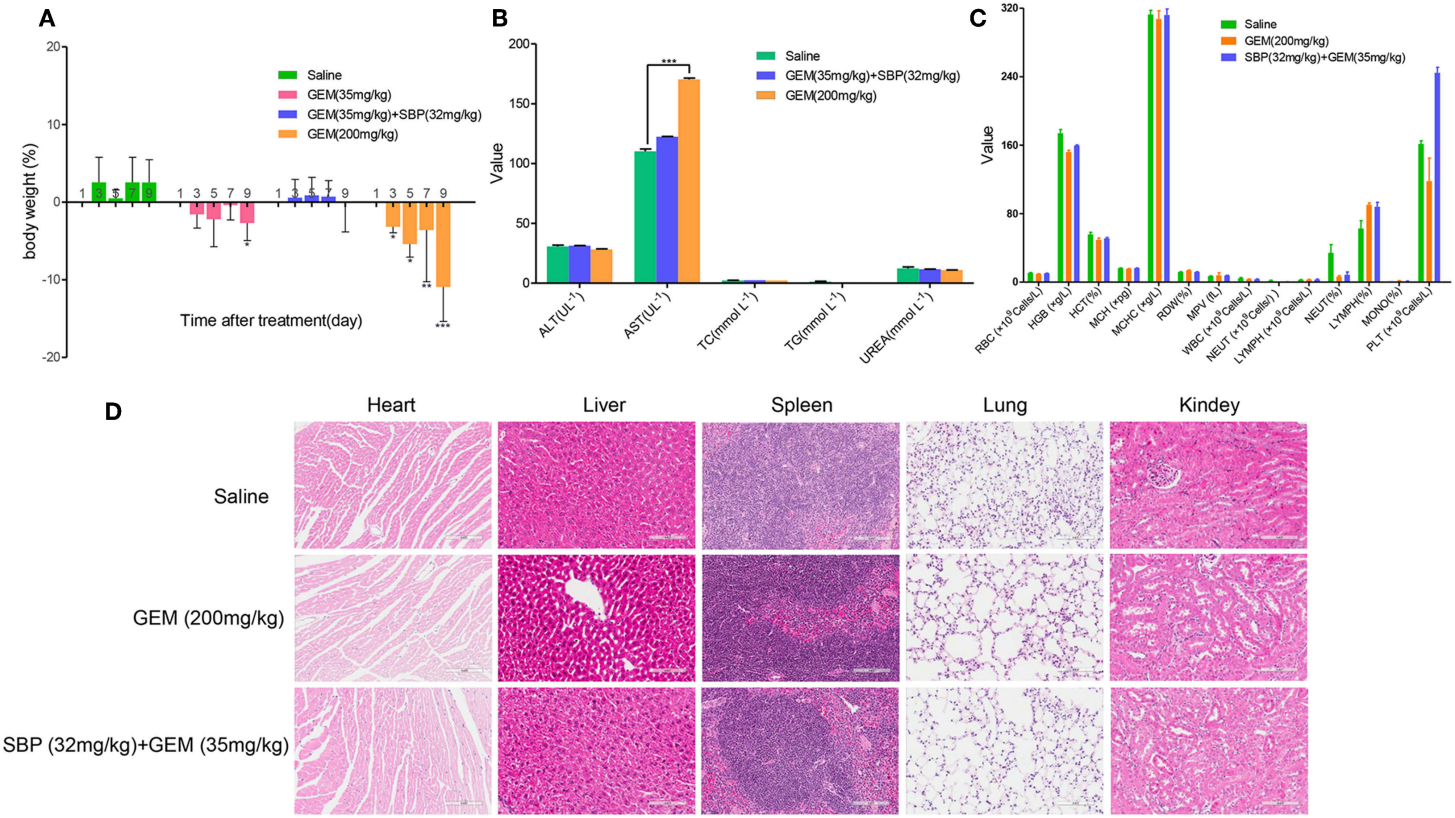
Minimizing side effects by using SBP combined with GEM. **(A)** Body weight changes of mice administrated with saline, GEM 35 mg/kg, GEM 200 mg/kg and SBP 32 mg/kg/GEM 35 mg/kg (*n* = 3–5 for each group). **(B,C)** Biochemical and hematological analysis of healthy mice treated with saline, GEM 200 mg/kg and SBP 32 mg/kg/GEM 35 mg/kg (*n* = 3–5 for each group). **(D)** Representative micrographs of H&E staining of mice organs.

Minimizing the toxic side effects is a crucial concern of improving cancer treatment. Although GEM is widely used clinically in cancer chemotherapy, its application is limited by the caused adverse effects, including anemia, leukopenia, neutropenia, thrombocytopenia, liver enzyme elevation (ALT, AST) among others(Katircibasi and Eken, 2017). Our results displayed that mice treated with GEM (200 mg/kg) significantly lost weight, however, administration of SBP (32 mg/kg)/GEM (35 mg/kg) had minimal effect on body weight (Figure 5A). Blood biochemistry analysis showed that administration of GEM (200 mg/kg) increased AST level dramatically while administration of SBP (32 mg/kg)/GEM (35 mg/kg) kept the AST at the normal level (Figure 5B, *P* < 0.001). Hematological analysis results and the representative H&E staining sections of major organs including heart, liver, spleen, lung and kidney were showed in Figures 5C,D. Hematological test and histological analysis of SBP (32 mg/kg)/GEM (35 mg/kg) did not show any changes compared to healthy mice.

### Antitumor Effects of SBP Combined With Other Chemotherapeutic Agents *in vivo*

**Figure 6:**
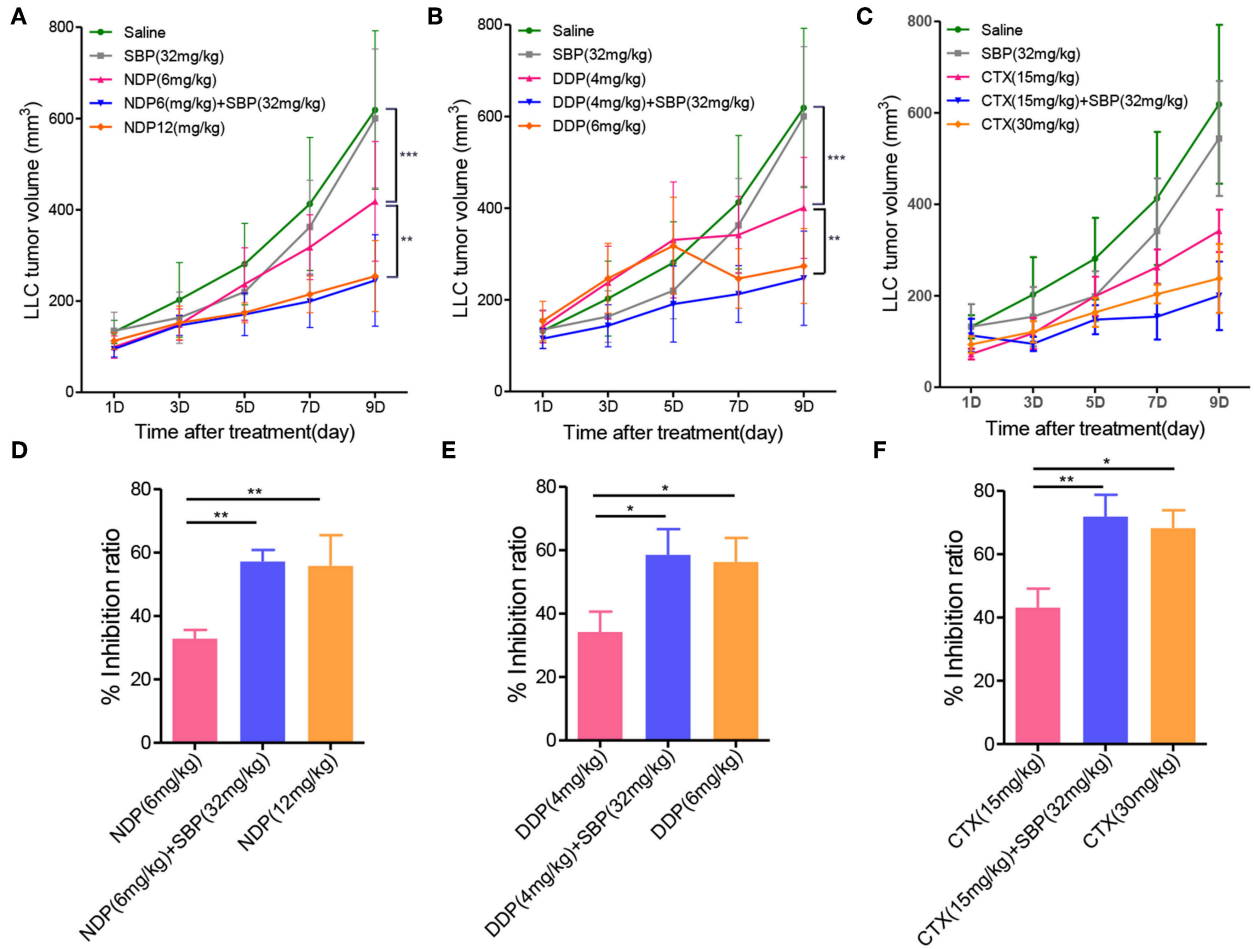
The effect of SBP combined with other chemotherapy drugs on tumor growth. **(A–C)** LLC tumor growth profile of mice treated with SBP (32 mg/kg) combined with NDP (6 mg/kg) or DDP (4 mg/kg) or CTX (15 mg/kg) in LLC tumor model. **(D–F)** Quantitative analysis of the tumor inhibition rate (*n* = 3–5 for each group). ^*^p < 0.05; ^**^p < 0.01.

In our continuation in the evaluation of whether or not the combination of SBP and other chemotherapeutic agents could have the same antitumor effect, we expanded the scope of anti-cancer drug by using NDP, DDP and CTX in combination with SBP to examine their antitumor effects *in vivo*. As shown in Figure 6, the tumor volume of the saline group on day 9 was more than 6-fold greater than on day 0, whereas the tumor volume of NDP (6 mg/kg), SBP (32 mg/kg)/NDP (6 mg/kg), DDP (4 mg/kg), SBP (32 mg/kg)/DDP (4 mg/kg), CTX (15 mg/kg), SBP (32 mg/kg)/CTX (15 mg/kg) on day 9 were 4-fold, 2.4-fold, 4-fold, 2.4-fold, 3.5-fold, <2-fold greater than on day 0, respectively. At treatment termination, the tumor inhibition rate of NDP (6 mg/kg), SBP (32 mg/kg)/NDP (6 mg/kg), DDP (4 mg/kg), SBP (32 mg/kg)/DDP (4 mg/kg), CTX (15 mg/kg), SBP (32 mg/kg)/CTX (15 mg/kg) were 32.80, 58.13, 34.15, 58.51, 44.71, and 68.35%, respectively. These data showed that similar effects were achieved when replacing Gem with NDP, DDP and CTX, indicating that combined use of SBP with other chemotherapeutic drugs can also enhance the antitumor effect.

## Discussion

Anti-angiogenesis has been recognized as an effective means of cancer treatment (Carmeliet and Jain, 2000). In the past few decades, many efforts have been made to reduce tumor vascular density in order to starving tumors. Unfortunately, this starving strategy may at the same time increase the level of tumor hypoxia and the possibility of drug resistance (Chen and Cleck, 2009; Jain, 2014). Inhibiting tumor vascular density and vascular function may be accompanied by limited drug delivery into tumor tissues, leading to failure to meet therapeutic expectations (Pavlidis and Pavlidis, 2013). The primary goal of intensive chemotherapy is to improve the delivery of drugs in tumors and new methods were developed to achieve that goal by enhancing blood perfusion. This strategy for treating tumors is radically different from previous methods and is called “vascular promotion therapy” which has been rarely studied (Bridges and Harris, 2015; Villanueva, 2015; Wong et al., 2015, 2016;Yin et al., 2017).

Cardiovascular diseases (CVD) and cancers remain the two leading causes of death in developed countries (Collaborators, 2017). Due to common risk factors for these diseases, many patients suffered from both cancers and CVD (Suter and Ewer, 2013). In addition, a number of tumor therapies are accompanied by the inherent cardiovascular toxicity, which may affect the prognosis and quality of life of cancer patients (Abi Aad et al., 2015). Thus, it is of significance for paying attention to interference between the two treatments.

TCMs have been employed to treat various diseases thousand years ago, and their effectiveness has also been confirmed in the literature (Tang et al., 2008). SBP is a famous TCM used for the treatment of cardiovascular diseases with a long history, which is composed of seven medicinal herbs with relative activities (Zhang et al., 2017; Dong et al., 2018). Recent reports suggest that this drug has pharmacological effects of anti-oxidation, endothelial protection and pro-angiogenesis efficiency (Li et al., 2015; Zhang et al., 2017; Guo et al., 2018; Yuan et al., 2018). Based on the above features of SBP, this drug was selected to be administered in combination with chemotherapeutic anti-cancer drugs in order to achieve synergistic effects.

Our *in vivo* results showed that the combination of SBP and GEM can actually improve cancer treatment efficiency and allow for lower doses of GEM to work as effectively as conventional higher doses. Then, in order to elucidate the mechanisms of this therapy, we focused on tumor angiogenesis. Our data demonstrated that by increasing tumor vascular density, blood perfusion, vascular permeability and tumor vessel dilation, co-used SBP could achieve better drug delivery efficiency. Furthermore, recent studies have shed light upon the critical role of tumor microenvironment for cancer progression, which is strongly correlated with cancer progression and chemoresistance (Martin et al., 2016; Gupta et al., 2017; Seleit, 2017). Angiogenesis has been reported to help in reducing tumor hypoxia (Wong et al., 2015). Angiogenesis is associated with modified tumor microenvironment, such as decreased tumor hypoxia and desmoplasia. We found that combining SBP and GEM enhanced oxygen supply and decreased the level of αSMA-positive myofibroblasts and collagen. Reducing toxic side effects is another important feature of enhancing tumor therapy. We showed that via enhancing the delivery and release of gemcitabine in tumors, we are able to minimize the adverse effects of the treatment whilst enhancing its efficacy. Finally, our data demonstrated that the combination of SBP and other chemotherapeutic agents can also improve the antitumor effect, suggesting that this treatment strategy could open a new way.

## Conclusion

**Figure 7:**
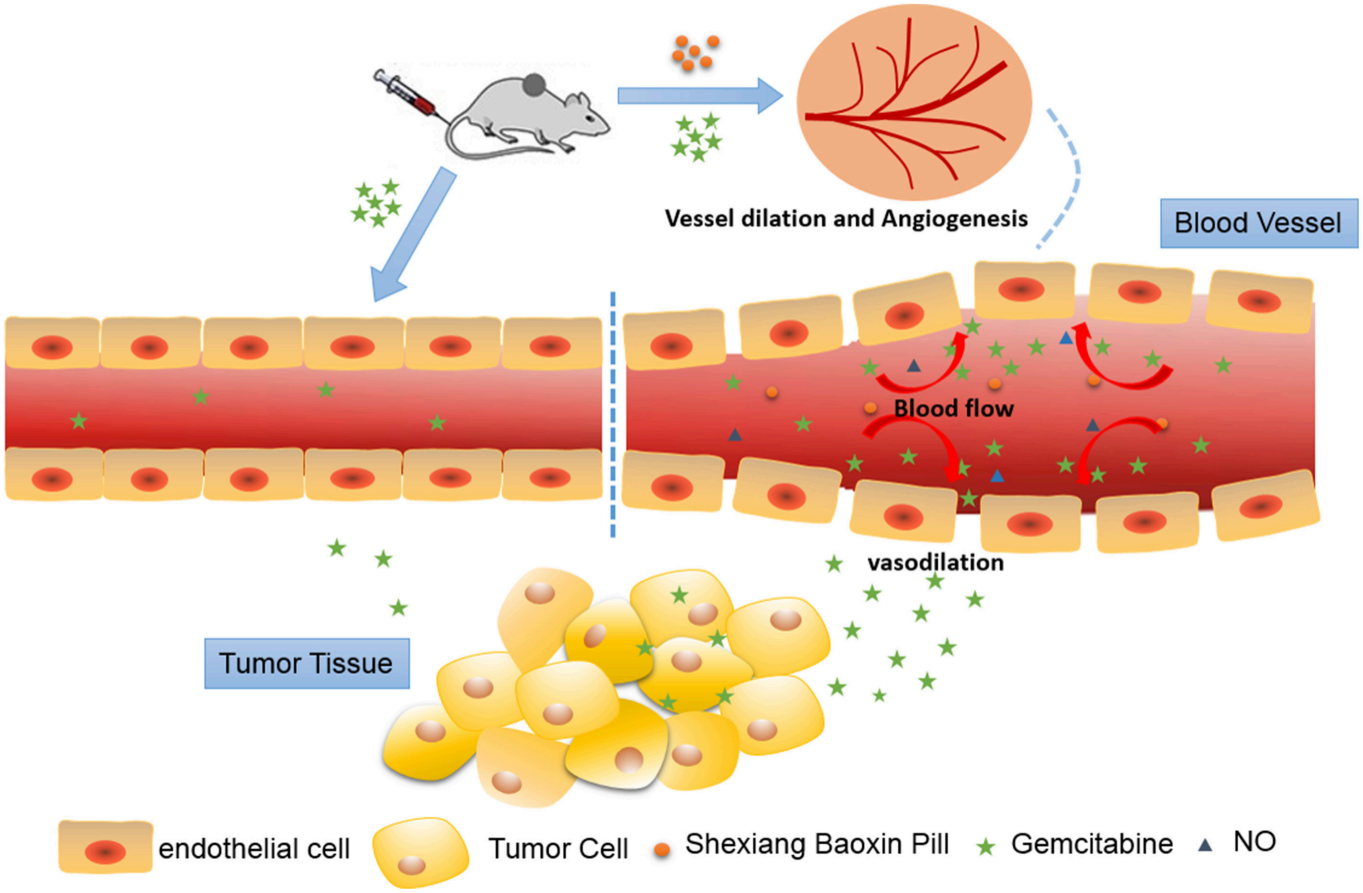
Schematic illustration of Shexiang Baoxin Pill increased tumor angiogenesis, blood perfusion and vessel dilation, which subsequently favored the delivery of Gemcitabine to the tumor lesion and modified tumor microenvironment.

In summary, our studies demonstrated that SBP could increase tumor angiogenesis, blood perfusion, vascular permeability and vasodilation, which facilitates the delivery of chemotherapeutic drugs to tumor (Figure 7). This is the first time to verify that SBP can be used in combination with anti-cancer drugs to promote the efficacy of cancer chemotherapy drugs, thereby improving tumor hypoxia and preventing drug resistance. This is the first report to apply SBP to tumor treatment, enriching the application range of SBP and possibly expanding clinical application.

## Data Availability

All datasets generated for this study are included in the manuscript and/or the supplementary files.

## Ethics Statement

This study was carried out in accordance with the recommendations of Committee for the Care and Use of Experimental Animals at Shanghai Institute of Traditional Chinese Medicine. The protocol was approved by the Shanghai Institute of Traditional Chinese Medicine.

## Author Contributions

JZ contributed to the conception and design of the study. LY, RL, XY, QC, and FW performed the experiments and analyzed the data. LY wrote the manuscript. GL gave advice on the writing. All authors have given approval to the final version of the manuscript.

### Conflict of Interest Statement

The authors declare that the research was conducted in the absence of any commercial or financial relationships that could be construed as a potential conflict of interest.
